# VIB1, a Link between Glucose Signaling and Carbon Catabolite Repression, Is Essential for Plant Cell Wall Degradation by *Neurospora crassa*

**DOI:** 10.1371/journal.pgen.1004500

**Published:** 2014-08-21

**Authors:** Yi Xiong, Jianping Sun, N. Louise Glass

**Affiliations:** Plant and Microbial Biology Department and The Energy Biosciences Institute, The University of California, Berkeley, Berkeley, California, United States of America; University College Dublin, Ireland

## Abstract

Filamentous fungi that thrive on plant biomass are the major producers of hydrolytic enzymes used to decompose lignocellulose for biofuel production. Although induction of cellulases is regulated at the transcriptional level, how filamentous fungi sense and signal carbon-limited conditions to coordinate cell metabolism and regulate cellulolytic enzyme production is not well characterized. By screening a transcription factor deletion set in the filamentous fungus *Neurospora crassa* for mutants unable to grow on cellulosic materials, we identified a role for the transcription factor, VIB1, as essential for cellulose utilization. VIB1 does not directly regulate hydrolytic enzyme gene expression or function in cellulosic inducer signaling/processing, but affects the expression level of an essential regulator of hydrolytic enzyme genes, CLR2. Transcriptional profiling of a Δ*vib-1* mutant suggests that it has an improper expression of genes functioning in metabolism and energy and a deregulation of carbon catabolite repression (CCR). By characterizing new genes, we demonstrate that the transcription factor, COL26, is critical for intracellular glucose sensing/metabolism and plays a role in CCR by negatively regulating *cre-1* expression. Deletion of the major player in CCR, *cre-1*, or a deletion of *col-26*, did not rescue the growth of Δ*vib-1* on cellulose. However, the synergistic effect of the Δ*cre-1*; Δ*col-26* mutations circumvented the requirement of VIB1 for cellulase gene expression, enzyme secretion and cellulose deconstruction. Our findings support a function of VIB1 in repressing both glucose signaling and CCR under carbon-limited conditions, thus enabling a proper cellular response for plant biomass deconstruction and utilization.

## Author Summary

Many filamentous fungi that grow on plant biomass are capable of producing lignocellulase enzymes to break down plant cell walls into utilizable sugars, thus holding great potential in reducing the cost of the next-generation biofuels. Cellulase production is subject to induction by the presence of plant biomass components and to repression by the availability of easily metabolized sugars, such as glucose. Genes required for repression of cellulase gene expression when preferred carbon sources are present (carbon catabolite repression) and those that play a role in mediating glucose sensing/metabolism have been identified in filamentous fungi, but the mechanisms involved in crosstalk between repression versus induction of cellulase gene expression is poorly understood. Here, we report the identification and functional characterization of VIB1, a transcription factor essential for plant cell wall deconstruction in *Neurospora crassa* and COL26, a transcription factor that functions in glucose sensing/metabolism and regulation of CCR. We show that disabling CRE1 repression and modulating the glucose response by deletion of *col-26* restored growth of the Δ*vib-1* mutant on cellulose. Our findings are particularly important in understanding the molecular basis of enzyme production that could allow a further strain improvement for plant biomass deconstruction.

## Introduction

Bioconversion of lignocellulosic biomass to simple sugars holds great promise in next-generation biofuel production and relies on a complex repertoire of proteins for enzymatic deconstruction of plant cell walls [1]. Many filamentous fungi have evolved to utilize cellulosic materials and are capable of producing a wide spectrum of enzymes, but only a few species have been harnessed for industrial usage [2]. Further improvement in fungal cellulolytic enzyme production is desired to make biofuel production cost-competitive, but this relies on a better understanding of the molecular basis of networks involved in carbon sensing and regulatory aspects associated with induction of gene expression of hydrolytic enzymes [3].

**Figure 1:**
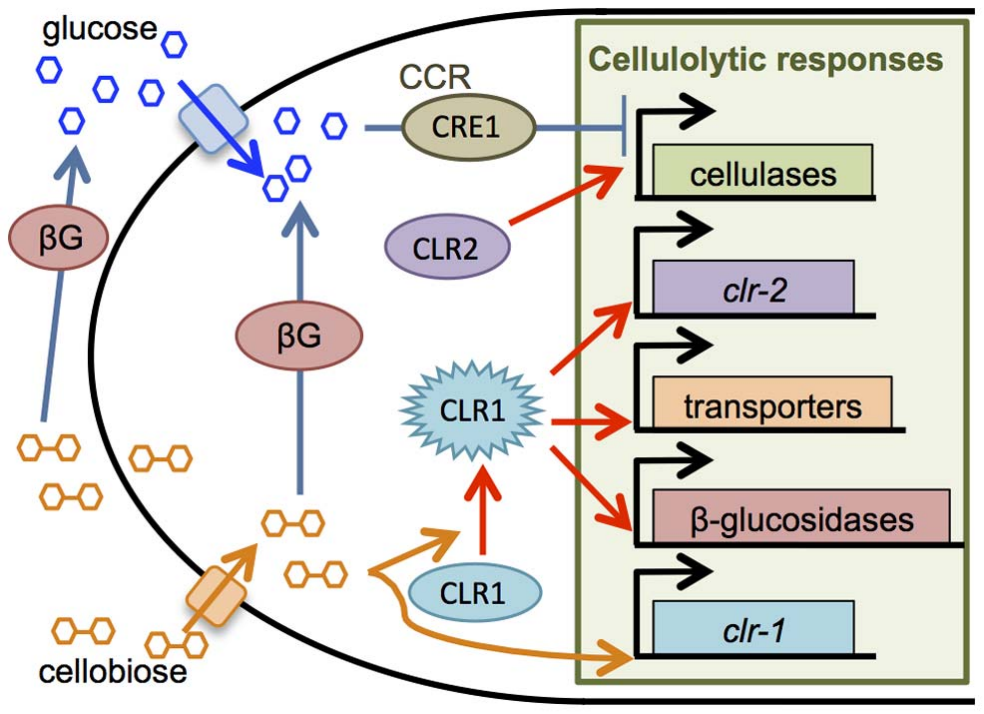
Cellulase production in *N. crassa* is regulated by cellobiose induction and CCR. CCR is decreased in absence of glucose, allowing scouting enzymes to liberate cellobiose from cellulose. Cellobiose (or a derivative) results in activation of the transcription factor CLR1, which induces expression of transporters for cellodextrins, β-glucosidases, and *clr-2*. Production of the transcription factor CLR2 drives cellulase gene expression. Both intracellular and extracellular β-glucosidase enzymes catalyze conversion of cellobiose to glucose, which can trigger carbon catabolite repression via glucose sensing mechanisms and transcriptional repression by CRE1.

Cellulolytic enzyme production and secretion is a unique attribute of filamentous fungi, and efforts to identify important factors in enzyme production led to the discovery of a number of transcriptional activators and repressors. For example, the transcription factor XlnR/XYR1 positively regulates expression of cellulase and hemicellulase genes in *Aspergillus niger* and *Trichoderma reesei*, respectively [4]–[7]. In *Neurospora crassa*, the transcription factors CLR1 and CLR2 are essential for growth on cellulose and are required for expression of a ∼212 gene regulon that is induced in response to cellodextrins, such as cellobiose [8], [9] (Figure 1). In *Aspergillus nidulans* and *A. oryzae*, a *clr-2* homolog, called *clrB/manR*, respectively, is also essential for cellulase gene expression and activity [8], [10], [11]. Additional transcriptional regulators that promote expression of some genes encoding hydrolytic enzymes have also been identified, including *mcmA* in *A. nidulans* [12], *clbR* in *A. aculeatus* [13], and *aceII* and *bglR* in *T. reesei* [14], [15].

In addition to induction, cellulase gene expression is also subject to carbon catabolite repression (CCR), which functions when a favorable carbon source, such as glucose, is present [3], [16], [17]. The most well-characterized transcription factor involved in CCR in filamentous fungi is CreA/CRE1. Deletion of *creA/cre-1* alleviates some aspects of CCR for cellulolytic enzyme expression in *Aspergilli* [18]–[22], *T. reesei* [23]–[25], *Penicillium decumbens* [2] and *N. crassa* [26], [27]. In *A. nidulans*, repression by CreA occurs both by binding to promoters of hemicellulase genes as well as repressing expression of transcriptional activators [28]. Other factors including *creB/cre2*, *creC*, *creD*, *lim1*, and *aceI* were also reported to promote CCR in different fungal species via unknown mechanisms [29]–[37]. The strength of CCR is tuned by glucose sensing and signaling, although crosstalk between these two regulatory systems is not well understood. In *N. crassa*, RCO3, a predicted sugar transporter was proposed to function as a glucose sensor [38], [39]. In *A. nidulans*, phosphorylation of glucose triggers CreA repression [29], [40]. In *Magnaporthe oryzae*, trehalose-6-phosphate synthase (Tps1) promotes glucose metabolism and CCR through inhibition of Nmr (nitrogen metabolite repression) proteins (Nmr1, Nmr2, Nmr3) [41]. Downstream, a multidrug and toxin extrusion pump, Mdt1, promotes citrate efflux to relieve CCR. To what extent these mechanisms are shared among cellulolytic fungi and whether they all converge to regulate CreA/CRE1-mediated CCR is currently unclear.

*N. crassa* is an early colonizer of burnt vegetation [42], [43], grows robustly on plant biomass and secretes a broad spectrum of enzymes to degrade plant cell walls [44], [45]. By screening the *N. crassa* near-full genome deletion strain set [46] for growth on Avicel (crystalline cellulose), we identified a transcription factor, *vib-1*, that is essential for cellulose utilization. VIB1 (vegetative incompatibility blocked) is a p53-like transcription factor that is conserved among filamentous ascomycete fungi. Characterized as a mediator of nonself recognition and cell death in *N. crassa* [47], [48], VIB1 is also required for extracellular protease secretion in response to both carbon and nitrogen starvation [48]. Here, we demonstrated that *vib-1* functions upstream of cellulolytic gene induction and its absence leads to a weak induction of *clr-*2 and cellulase genes but increased expression of genes predicted to function in CCR. Functional analysis of one such predicted transcription factor gene, *col-26*, an *N. crassa bglR* homolog, showed that COL26 regulates glucose sensing/metabolism and which is separate from CRE1-mediated CCR. Deletion of both *col-26* and *cre-1* leads to a synergistic effect in rescuing Δ*vib-1* utilization of cellulose and cellulolytic activity. Our data support a function for VIB1 in repression of glucose signaling and CCR and which is critical for fungal utilization of plant biomass.

## Results

### Deletion of *vib-1* causes a growth defect on cellulosic biomass

**Figure 2:**
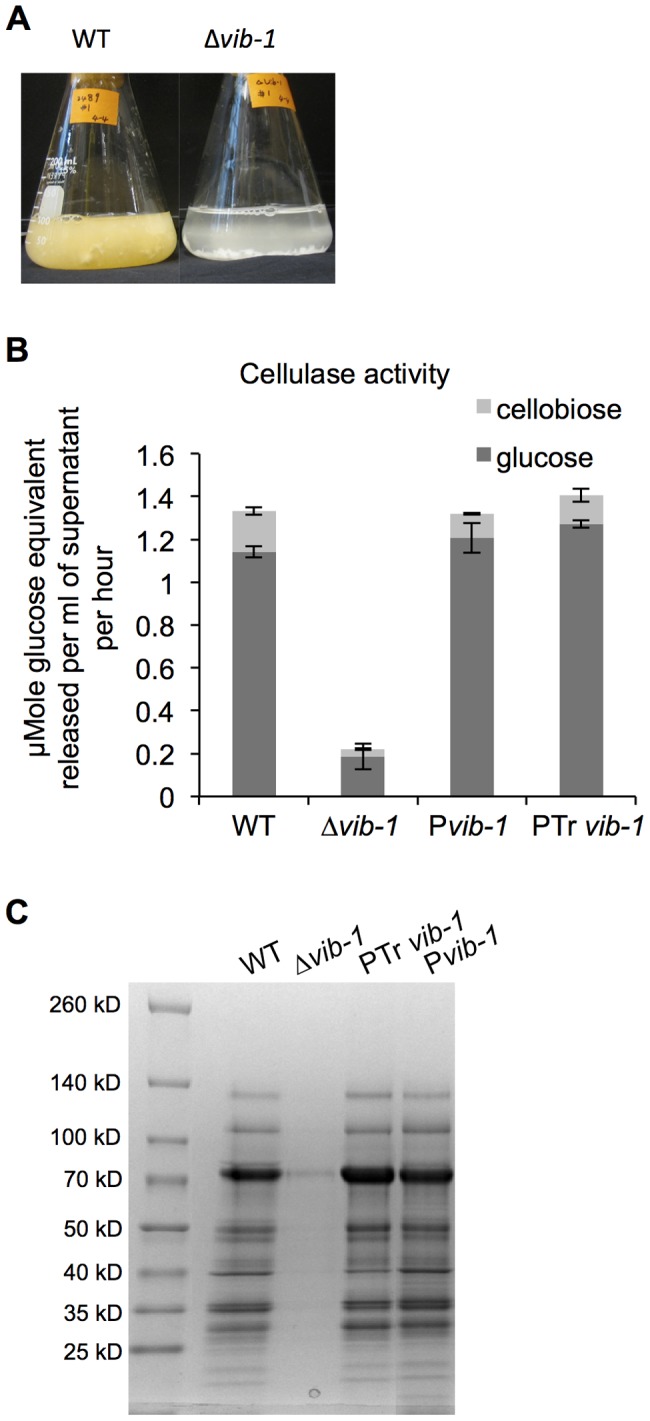
Deletion of *vib-1* abolishes production of cellulases and utilization of cellulosic material. (A) Growth of WT and Δ*vib-1* on Avicel after 4 days; growth of WT is indicated by formation of orange mycelia, versus no growth of the Δ*vib-1* mutant. (B) Cellulase activity from 4-day old culture supernatants from Avicel-grown cultures of WT, the Δ*vib-1* mutant, the P*vib-1* strain (constitutive expression of *vib-1* in a Δ*vib-1* strain) and the P*Trvib1* strain (constitutive expression of *T. reesei vib1* in a Δ*vib-1* strain). Cellulase activity was measured using Avicel as a substrate and represented by the amount of glucose and cellobiose released. The equivalent of glucose from cellobiose was calculated and represented by the light gray bar. (C) The secretomes of strains analyzed in panel B are shown.

Screening of a transcription factor deletion set of *N. crassa* strains [46] for ability to deconstruct crystalline cellulose showed that a strain carrying a deletion of the *vib-1* gene (FGSC11309) failed to grow on Avicel (Figure 2A). Since functional *vib-1* is required for extracellular protease secretion in response to carbon and nitrogen starvation in *N. crassa* [48], [49], we hypothesized that the Δ*vib-1* mutant might be unable to respond to complex extracellular carbon sources. In support of this hypothesis, the Δ*vib-1* mutant also exhibited slow growth on xylan. Growth defects were accompanied by barely detectable extracellular enzyme activity towards crystalline cellulose and low extracellular xylanase activity (Figure 2B and S1A). In contrast, the Δ*vib-1* mutant accumulated a similar amount of mycelial biomass as the WT strain when inoculated into minimal media containing simple sugars (sucrose, cellobiose or xylose) (Figure S1B). The introduction of an ectopic copy of *vib-1* (*Pvib-1*) completely restored the growth defects on Avicel of the Δ*vib-1* mutant, as well as the secretome and cellulolytic enzyme activity of culture supernatants (Figure 2B).

To test the hypothesis that the role of VIB1 in cellulose utilization is conserved in other filamentous fungi, especially in fungi used in industrial production of cellulolytic enzymes, we carried out complementation tests using the *vib-1* ortholog from *T. reesei* (EGR52133; *Trvib1*); TrVIB1 and *N. crassa* VIB1 share 49% amino acid identity. Constitutive expression of *Trvib1* in a *N. crassa* Δ*vib-1* mutant fully restored the growth and cellulolytic enzyme activity (Figure 2B). The *Trvib1* strain also recapitulated most of the secretome of *N. crassa* WT and *Pvib-1* strains on Avicel (Figure 2C). These results suggest that *vib-1* is functionally conserved for the utilization of cellulose in filamentous ascomycete fungi.

The Δ*vib-1* mutant shows an inappropriate temporal and spatial conidiation pattern. These phenotypes are correlated with differential localization of VIB1-GFP in vegetative hyphae versus conidiophores [48]. As conidiation is regulated by glucose limitation [50], we assessed whether differential localization of VIB1 was also associated with cellulose utilization. We examined a strain in which we replaced the resident *vib-1* gene with a functional *vib-1-gfp* construct. Nuclear localization of VIB1-GFP was observed in hyphae and localization was independent of carbon source, either following a shift to Avicel for 2 hrs (Figure S1C) or after prolonged growth.

### Constitutive expression of *clr-2* restores cellulose utilization in the Δ*vib-1* mutant

Previous comparative RNA-seq analysis of WT revealed that 212 genes are significantly differentially expressed under cellulose conditions, a gene set referred to as the “Avicel regulon” [8]. To determine whether the defect in cellulase secretion and activity in the Δ*vib-1* mutant was due to failure to induce cellulase gene expression versus a defect in cellulase secretion, we assessed genome wide expression differences via RNA-seq between the WT and Δ*vib-1* strains following a shift for 4 hrs from sucrose medium to either carbon-free or Avicel medium. Of the 212 genes in the Avicel regulon, 91 genes were expressed at a significantly lower level in the Δ*vib-1* mutant versus WT under Avicel conditions (cutoff: Padj <0.05 and fold change >2; Table S1). This gene set includes the essential cellulase transcription factor gene, *clr-2* and 43 carbohydrate-active enzymes (CAZy) from 27 different families (Carbohydrate Active Enzymes database: http://www.cazy.org/) [51].

**Figure 3:**
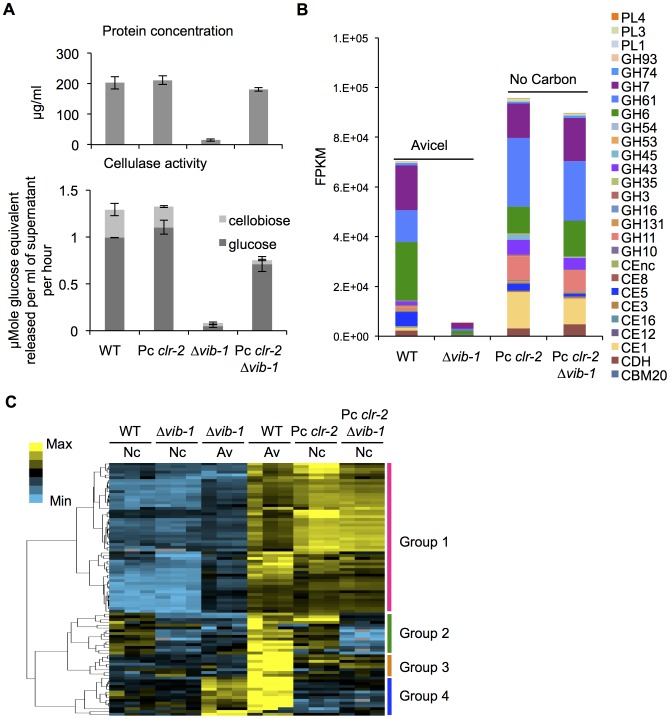
Constitutive expression of *clr-2* rescued the cellulase production defect of the Δ*vib-1* mutant. (A) Protein concentration and cellulase activity in a Δ*vib-1* mutant versus a Δ*vib-1* strain constitutively expressing *clr-2* (Pc *clr-2*; Δ*vib-1*) and WT and a Pc *clr-2* strain under Avicel conditions. (B) Expression levels from RNA-seq data of genes encoding major classes of CAZy proteins from WT and Δ*vib-1* shifted to Avicel versus the Pc *clr-2* and Pc *clr-2*; Δ*vib-*1 strains shifted to minimal media with no carbon source. FPKM (Fragment Per Kilobase per exon per Megabase mapped) for individual genes were averaged between three biological replicates and pooled by CAZy class. (C) Hierarchical clustering of FPKM for 91 Avicel-regulon genes in the Δ*vib-1* mutant and WT on Avicel (Av) and the Δ*vib-1*, WT, Pc *clr-2* and the Pc *clr-2*; Δ*vib-*1 strains switched to no carbon conditions (Nc). Results are displayed as heat maps with log (FPKM) from minimum (bright blue) to maximum (bright yellow).

CLR1 and CLR2 are strictly required for full expression of 140 genes within the Avicel regulon [8], [10]; 62 of these genes were identified in the 91-gene set that showed low expression in the Δ*vib-1* mutant. Although expression of *clr-1* was not significantly different from WT in the Δ*vib-1* mutant, the expression of *clr-2* was significantly reduced (FPKM: 107±43 in WT; 66±27 in Δ*vib-1* for *clr-1* versus 171±10 in WT; 39±14 in Δ*vib-1* for *clr-2*) (Table S1). Importantly, constitutive expression of *clr-2* (Pc *clr-2*) in minimal medium without cellulosic inducers recapitulates the response of *N. crassa* to crystalline cellulose, including the secretion of active cellulolytic enzymes [10]. The reduced transcription of *clr-2* in the Δ*vib-1* mutant (Table S1) suggested that constitutive expression of *clr-2* might suppress the cellulose utilization defect in the Δ*vib-1* mutant. To test this hypothesis, we constructed a Pc *clr-2*; Δ*vib-1* strain and evaluated its ability to secrete cellulases and utilize Avicel in comparison to the Pc *clr-2*, the Δ*vib-1* and WT strains. In support of our hypothesis, the Pc *clr-2*; Δ*vib-1* strain showed restoration of protein secretion and cellulolytic activity to near WT levels (Figure 3A). Although the *clr-2* expression levels in the Pc *clr-2* strain were at a similar level to a WT strain after a 4 hr shift to Avicel (Figure S4), the Pc *clr-2*; Δ*vib-1* mutant showed a ∼3.5 fold increase in *clr-2* expression level under the same conditions.

To evaluate the functions of VIB1 versus CLR2 in regulating the 91 genes in the Avicel-regulon, we generated RNA-seq data from the Pc *clr-2*; Δ*vib-1* mutant that was shifted for 4 hrs from sucrose medium to carbon-free medium and compared it to previously obtained data for Pc *clr-2* [10]. Analysis of genes encoding different CAZy family proteins revealed a similar pattern of expression between the Pc *clr-2* and the Pc *clr-2*; Δ*vib-1* strains (Figure 3B), consistent with our hypothesis that VIB1 functions upstream of *clr-2* in response to cellulose. To differentiate whether any Avicel-regulon genes that showed decreased expression levels in Δ*vib-1* mutant were due to the *vib-1* deletion rather than a low level of *clr-2* expression, we performed hierarchical clustering of expression patterns of the Avicel-regulon genes across 6 RNA-seq experiments (WT and Δ*vib-1* shifted to no carbon or Avicel and the Pc *clr-2* and the Pc *clr-2*; Δ*vib-1* strains shifted to no carbon); 4 major expression groups were identified (Figure 3C and Table S1). Two groups (group 1 and 3) were CLR2-regulon genes that were *vib-1* independent. Group 1 consisted of 54 genes whose expression was fully induced by constitutive expression of *clr-2* regardless of the presence or absence of *vib-1*, including 33 of the 43 CAZy proteins (Table S1). Group 3 consisted of 8 genes whose expression was partially induced by *clr-2*, but still in a *vib-1*-independent manner. The fourth group of genes included 14 *vib-1* modulated genes. These genes were partially induced in Δ*vib-1* on Avicel, but remained repressed in both the Pc *clr-2* and the Pc *clr-2*; Δ*vib-1* strains under no carbon conditions (Table S1). Expression of these genes is likely induced by the cellulolytic cascade pathways upstream of CLR2 or other components present in commercial Avicel preparations, such as a low concentration of hemicellulose [9]. The second group consisted of 15 genes that were induced by constitutive *clr-2* expression under no carbon conditions but in *vib-1-*dependent manner. This gene set included a pectate lyase (NCU06326), a BNR/Asp-box repeat protein predicted to have exo-α-L-1,5-arabinanase activity (NCU09924), a β−xylosidase (NCU09923/*gh3-7*), an extracellular β−1,4-D-glucosidase (NCU04952/*gh3-4*), a β−1,3-glucosidase (NCU09904), a starch binding domain-containing protein (NCU08746), a LysM domain-containing protein (NCU05319), a putative methyltransferase (NCU05501), and 6 hypothetical proteins. Six genes in this set encode proteins predicted to enter the secretory pathway (Table S1).

### VIB1 functions upstream of the inducer signal

**Figure 4:**
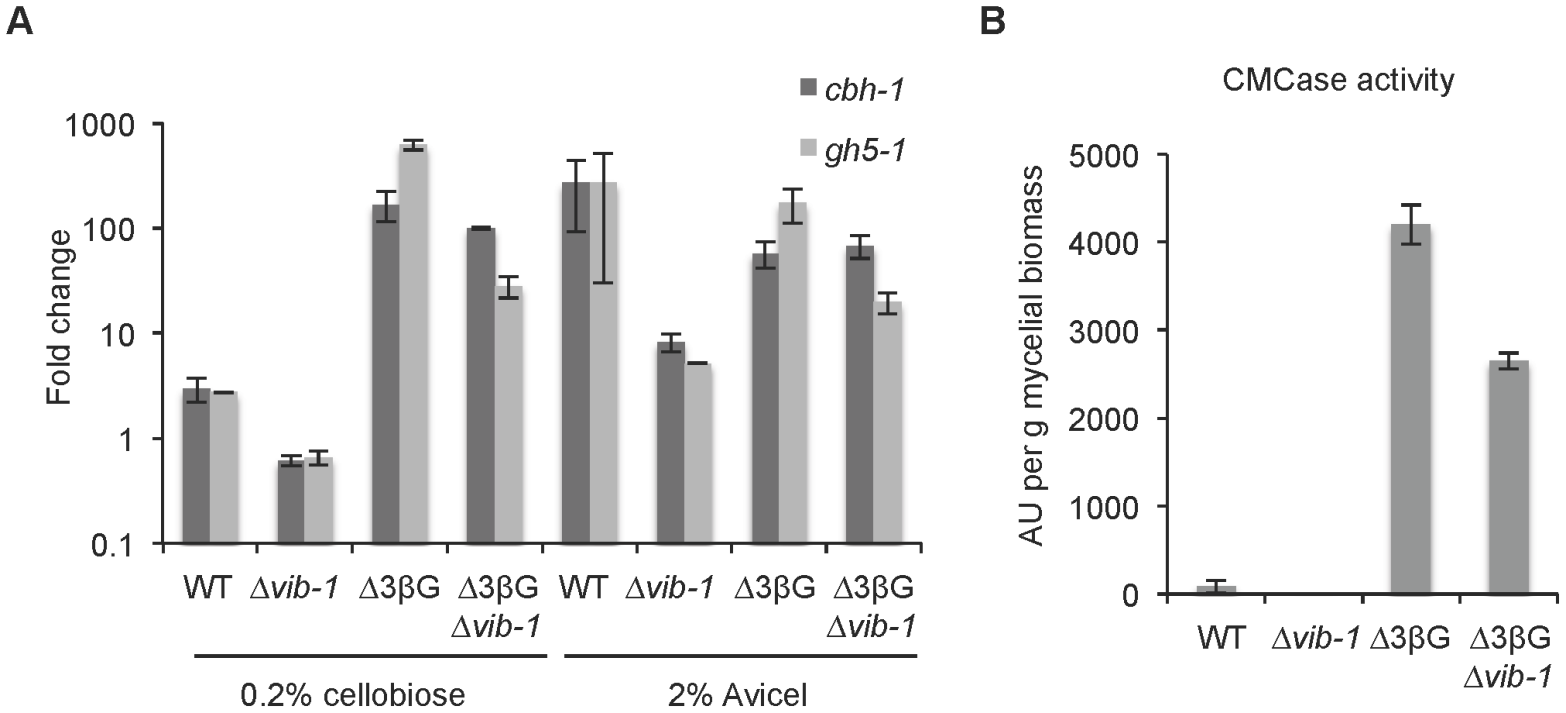
VIB1 is not required for cellobiose sensing or signaling. (A) Expression levels of two cellulase genes (*cbh-1* and *gh5-1*) were assessed in the Δ3βG; *Δvib-1* strain versus WT, and the Δ3βG and *Δvib-1* strains after a shift from sucrose VMM to 0.2% cellobiose versus 2% Avicel. Gene expression levels were measured by relative quantitative-PCR using actin as a control and normalized to expression level when cultures were shifted to VMM with no carbon source. (B) CMCase activity after 24 hrs of growth on 2% cellobiose of the Δ3βG; *Δvib-1* strain relative to the WT, and the Δ3βG and *Δvib-1* strains. Measured enzyme activity in arbitrary unit (AU) was normalized against to the mycelial biomass of each culture.

Our epistasis experiments indicated that *vib-1* functions upstream of *clr-2*, suggesting that VIB1 could be involved in signal molecule processing that leads to CLR1 activation and thus *clr-2* expression (Figure 1). In *N. crassa*, a strain carrying deletions of genes encoding two extracellular β-glucosidases and an intracellular β-glucosidase (Δ3βG), recapitulates the cellulolytic response when the Δ3βG strain is exposed to cellobiose [9]. These data indicate that cellobiose (or a derivative) functions as a cellulose signal that results in the induction of cellulolytic genes and subsequent secretion of cellulase enzymes. This cellobiose-induced cellulase gene expression and secretion is dependent upon functional *clr-2* gene, as the Δ3βG; Δ*clr-2* mutant is unable to produce cellulolytic enzymes in response to Avicel or cellobiose (unpublished data). We therefore asked if VIB1 plays a role in induction via signal processing. To test this hypothesis, we created a Δ3βG; Δ*vib-1* quadruple mutant and asked whether the Δ3βG; Δ*vib-1* mutant could induce cellulase gene expression in response to cellobiose. Following a switch from sucrose to either no carbon, or 0.2% cellobiose, or 2% Avicel for 4 hrs, the induction of two major cellulase genes, *cbh-1*/NCU07340 and *gh5-1*/NCU00762 were significantly induced in the Δ3βG; Δ*vib-1* and the Δ3βG strains, but not in the Δ*vib-1* strain (p<0.05)(Figure 4A).

The restoration of cellulase gene expression in the Δ3βG; Δ*vib-1* strain when exposed to cellobiose was accompanied by enzyme production and activity. Similar to the Δ3βG mutant, the Δ3βG; Δ*vib-1* strain accumulated biomass more slowly on cellobiose than WT or the Δ*vib-1* mutant due to the slow conversion of cellobiose to glucose (0.51±0.11 g/L and 0.63±0.0 g/L for the Δ3βG and the Δ3βG; Δ*vib-1* strains, respectively, versus 3.83±0.19 g/L and 3.62±0.11 g/L for WT and Δ*vib-1*, respectively). However, despite less biomass accumulation, both the Δ3βG and the Δ3βG; Δ*vib-1* strains showed significantly more enzyme activity than WT and the Δ*vib-1* strains on 2% cellobiose (Figure 4B). When grown on medium containing 2% Avicel as a sole carbon source, the Δ3βG; Δ*vib-1* strain showed significantly higher enzyme activity than Δ*vib-1* (Figure S2). These expression and activity data indicate VIB1 does not play a role in signal processing or signal transduction mechanisms that lead to activation of CLR1 and transcription of the cellulase activator, CLR2.

### Comparative analysis of transcriptomes of WT and the Δ*vib-1* strain

**Figure 5:**
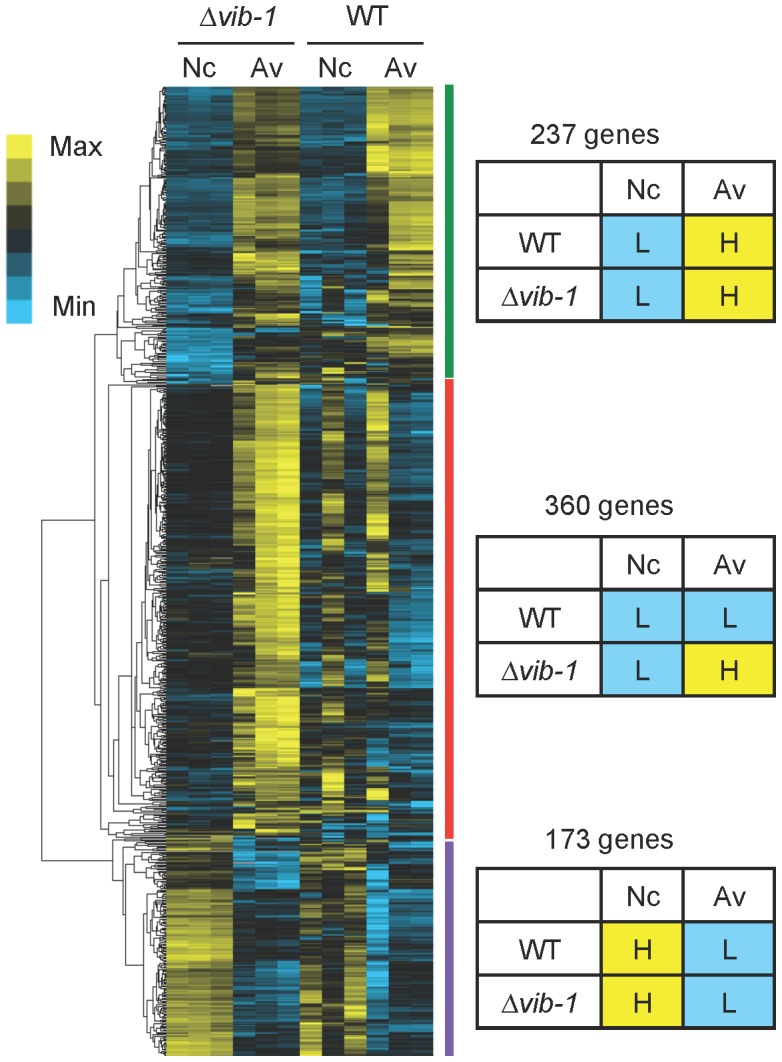
Comparative analysis of gene expression between Δ*vib-1* and WT shifted to media lacking a carbon source (Nc) versus Avicel (Av) revealed potential CCR regulators. Hierarchical clustering of FPKM for 770 genes that were differentially expressed in the Δ*vib-1* mutant when shifted to either carbon-free and Avicel conditions. Heat maps showing log (FPKM) from minimum (bright blue∶L) to maximum (bright yellow∶H) revealed three gene sets: A 273-gene set that has a similar expression pattern between the Δ*vib-1* mutant and WT, although the induction level on Avicel differed; a 173-gene set that displayed a similar expression pattern between the Δ*vib-1* mutant and WT that is high (H) on Nc and low (L) on Avicel; a 360-gene set consisting of genes that were generally expressed at higher levels in *Δvib-1* on Avicel than in other samples/conditions.

In addition to induction, cellulolytic enzyme production requires proper nutrient sensing and relief from carbon catabolite repression (CCR) (reviewed in [17], [52]). We therefore hypothesized that the Δ*vib-1* mutant might be defective in either nutrient sensing and/or relieving CCR in response to Avicel. To test this hypothesis, we first compared RNA-seq data of the Δ*vib-1* mutant when shifted from sucrose to carbon-free media versus a shift from sucrose to Avicel media. This comparison revealed 770 differentially expressed genes (cutoff: Padj<0.01 and fold change >2) (Table S2). We then compared how these genes were expressed in WT under no carbon versus Avicel conditions using a previously published RNA-seq dataset [8]. Hierarchical clustering analysis of expression patterns of these 770 genes revealed three gene clusters (Figure 5) (Table S2).

The first cluster contained 237 genes whose expression pattern was similar between the Δ*vib-1* and WT strains. This gene set was expressed at low levels under no carbon conditions but induced to higher levels upon exposure to Avicel. This group contained 51 CAZy proteins, *clr-1* and *clr-2*, all three cellodextrin transporters (*cdt-1*, *cdt-2*, and *cbt-1*) [44], [53], [54] and 102 hypothetical proteins. This gene set overlapped the WT Avicel-regulon for 143 genes, suggesting that cellulosic induction still occurred in the Δ*vib-1* mutant albeit at a low level.

The second cluster consisted of 173 genes whose expression pattern was also similar between WT and the Δ*vib-1* strain. However, in contrast to the first gene set, the expression level of these 173 genes was higher under carbon-free conditions. This set included 7 CAZy proteins, three conidiation-specific proteins (NCU08769/*con-6*, NCU07325/*con-10*, NCU09235/*con-8*), a high affinity glucose transporter/NCU08152, and 103 hypothetical proteins. Genes in this cluster may encode proteins that function in a general response to carbon starvation.

The third cluster consisted of 360 genes whose expression pattern between no carbon and Avicel conditions was different in the Δ*vib-1* mutant as compared to the WT strain. This gene set showed consistently higher expression in the Δ*vib-1* mutant on Avicel medium as compared to carbon-free medium (Figure 5). Only 7 genes encoding CAZy proteins were in this set and 169 genes were annotated as hypothetical. An enrichment in the categories of metabolism and energy, particularly, degradation of glycine (p = 2.37e-03), nitrogen, sulfur and selenium metabolism (p = 8.00e-03), purine nucleotide/nucleoside/nucleobase catabolism (p = 2.49e-05), isoprenoid metabolism (p = 8.63e-04), respiration (p = 3.34e-04), metal binding (p = 6.18e-04), and mitochondrial transport (p = 2.94e-03) was observed. These data suggested that the Δ*vib-1* mutant was improperly responding to carbon-limited conditions as compared to a WT strain.

Within the gene set that showed increased expression level in the Δ*vib-1* mutant on Avicel were genes involved in CCR. This gene set included *cre-1*/NCU08807, *creD*/NCU03887, *creB*/NCU08378 and *bglR*/NCU07788 (Table S2). Although the role of *cre-1* in CCR and cellulose utilization is established in *N. crassa* [26], [27], the function of the *creB* and *creD* homologs in cellulolytic enzyme production were uncharacterized. In *N. crassa*, NCU07788/*BglR* was previously characterized in a transcription factor deletion screen and was named *col-26* for its colonial phenotype on minimal sucrose medium [46].

### The identification and characterization of new proteins involved in carbon sensing

**Figure 6:**
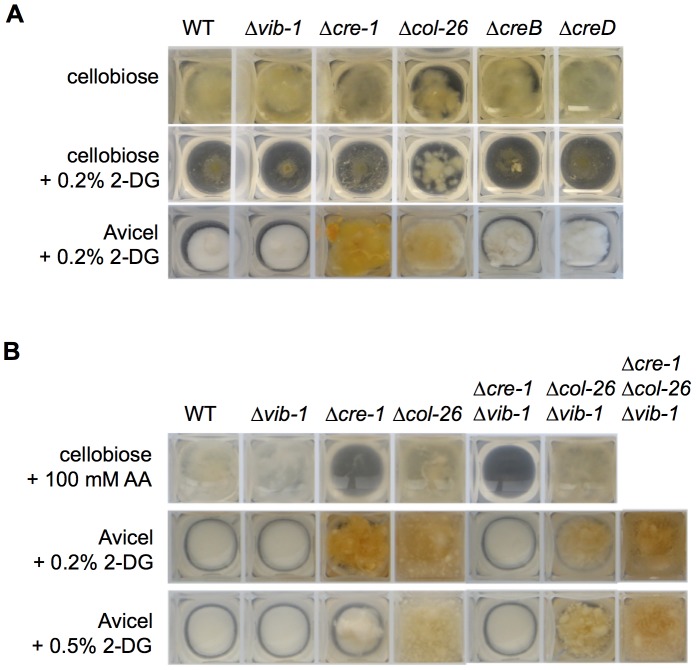
Screen for function of new proteins involved in CCR. (A) Growth assays of the WT, *Δvib-1*, Δ*cre-1*, Δ*col-26*, Δ*creB*, and Δ*creD* strains on 2-deoxy-glucose (2-DG) when grown on 2% cellobiose VMM for 2 days or on 2% Avicel VMM for 4 days. (B) Effects of *col-26* and *cre-1* deletions on sensitivity to 2-DG and allyl alcohol. Strains were inoculated and grown in 2% cellobiose VMM with 100 mM allyl alcohol for 40 hrs. For 2-DG sensitivity tests, the strains were inoculated and grown in 2% Avicel with either 0.2% 2-DG or 0.5% 2-DG for 5 days.

To determine whether homologs of the CCR genes that showed increased expression in the *Δvib-1* mutant play a role in cellulose deconstruction, we first measured protein concentration and cellulase enzyme activity in supernatants from the Δ*col-26*, ΔNCU08378/*creB*, and ΔNCU03887/*creD* mutants grown on Avicel for 7 days: none of the mutants showed significantly different cellulase activity than WT (Figure S3). To test if these genes are involved in CCR, we evaluated resistance of WT and the mutants to 2-deoxy-glucose (2-DG). The compound 2-DG is an analogue of glucose that cannot be metabolized and is often used to select for, or evaluate, impairment of CCR and glucose repression in filamentous fungi [39], [55]–[57]. In strains with functional CCR, 2-DG is phosphorylated, thus activating CCR, resulting in the inability of the strain to grow on alternative carbon sources; strains with impaired CCR are insensitive to 2-DG exposure. When 2% cellobiose and 0.2% 2-DG were used as carbon sources, only the Δ*cre-1* and the Δ*col-26* mutants showed 2-DG resistance, which was more obvious when Avicel instead of cellobiose was used as a carbon source (Figure 6A). These data implicated COL26 in CCR in *N. crassa*.

**Figure 7:**
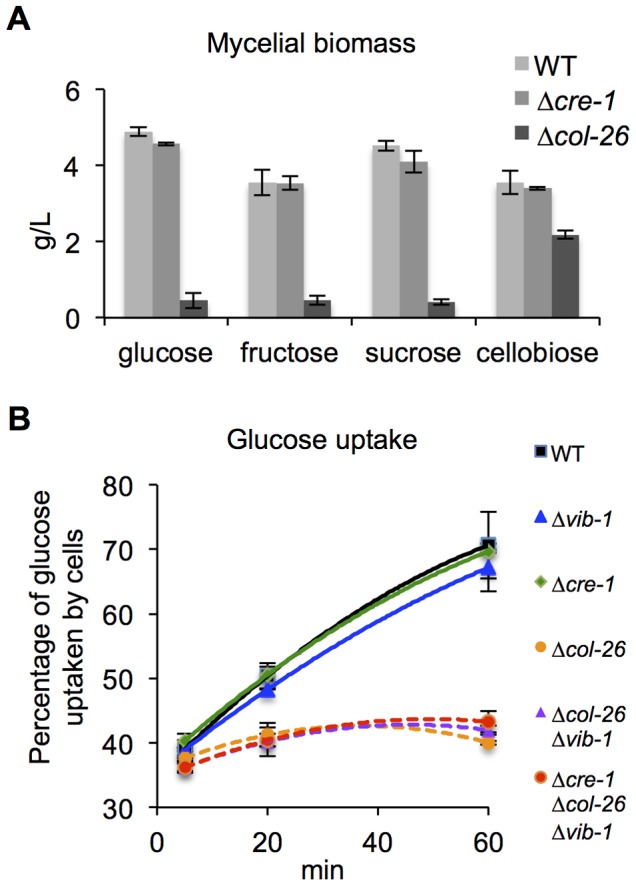
Deletion of *col-26* causes defects in glucose sensing/metabolism. (A) Mycelial biomass of the Δ*col-26* mutant relative to WT and the Δ*cre-1* strains on glucose, fructose, sucrose, or cellobiose. Mycelial biomass was measured at 24 hrs after inoculation. (B) Glucose uptake of WT, Δ*vib-1*, Δ*cre-1*, Δ*col-26*; Δ*vib-1* and the Δ*cre-1*; Δ*col-26*; Δ*vib-1* mutants was assayed by monitoring glucose remaining in the medium at 5 min, 20 min, and 60 min from cultures of identical biomass.

To confirm the role of COL26 in CCR, we tested CCR functionality using allyl alcohol (AA). As reported for *M. oryzae* [41], when CCR is impaired, alcohol dehydrogenase is expressed and will convert AA into toxic acrylaldehyde. Thus, strains with impaired CCR exhibit AA sensitivity, while strains with functional CCR are insensitive. As predicted, the Δ*cre-1* mutant was sensitive to AA, but the Δ*col-26* mutant, similar to WT, was insensitive (Figure 6B). These data indicated that CCR was still functional in the Δ*col-26* mutant. To reconcile the different results for the Δ*col-26* mutant with respect to CCR, we analyzed growth of the Δ*cre-1* and the Δ*col-26* mutants on different simple carbon sources. When grown on MM media with 2% glucose, fructose, sucrose, or cellobiose as the sole carbon source, the Δ*cre-1* mutant accumulated a similar amount of biomass to the WT strain (Figure 7A). However, the Δ*col-26* mutant exhibited a severe growth defect on glucose, fructose and sucrose, consistent with its colonial designation [46], but only a moderate growth defect on cellobiose (Figure 7A).

The fact that the Δ*col-26* mutant grew much better on cellobiose as compared to glucose, fructose, and sucrose and was insensitive to 2-DG suggested that the Δ*col-26* mutant might have defects in sugar transport and/or metabolism. To test this hypothesis, we measured glucose uptake rates in WT, the Δ*cre-1*, and the Δ*col-26* mutants. Within the first 5 minutes, extracellular glucose was reduced to a similar level in all strains (Figure 7B), suggesting similar glucose transporting capacity. However, over the remaining 55 minutes, glucose uptake rates decreased dramatically in the Δ*col-26* mutant (Figure 7B). These data indicate that the Δ*col-26* mutant has defects in glucose sensing/metabolism, rather than in glucose transport.

### Simultaneous inhibition of glucose sensing/metabolism and impairment of CRE1-mediated CCR rescues Δ*vib-1* growth on Avicel

**Figure 8:**
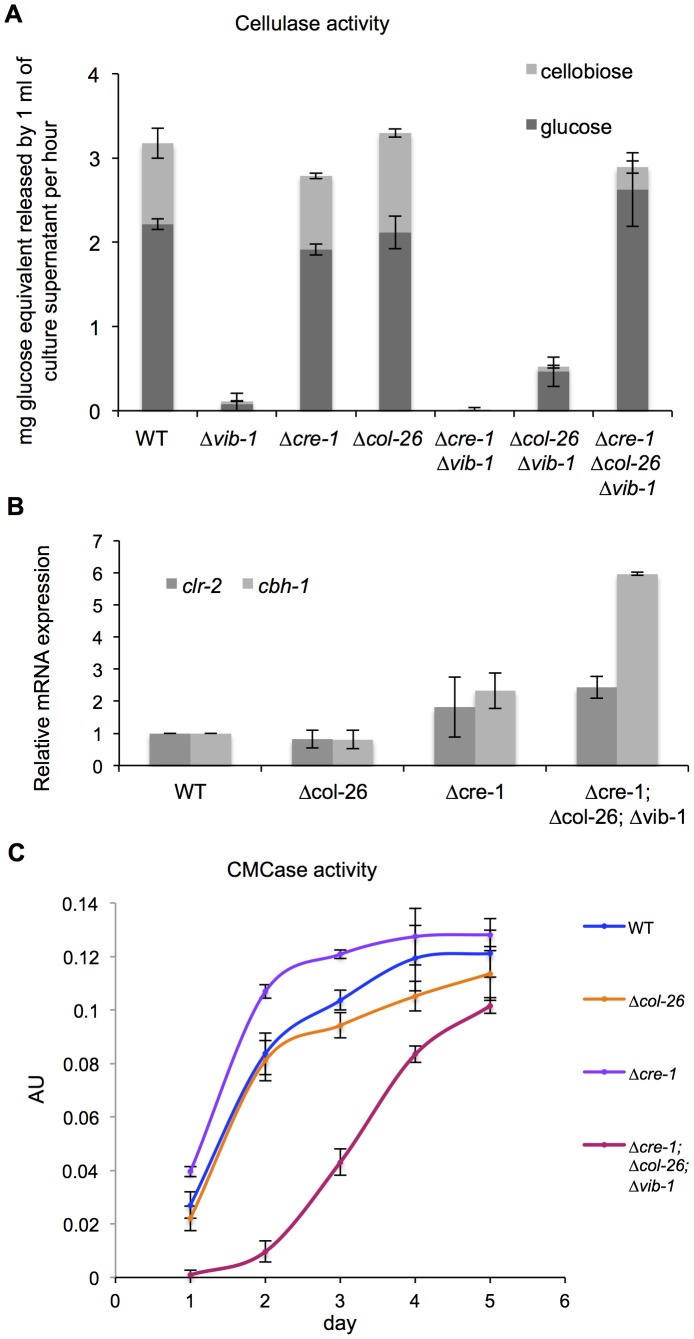
Simultaneous deletion of *cre-1* and *col-26* rescues the phenotype of Δ*vib-1* on cellulose. (A) Cellulase activity of culture supernatants after 4-days of growth on Avicel from WT versus Δ*vib-1*, Δ*cre-1*, Δ*col-26*, Δ*cre-1*; Δ*vib-1*, Δ*col-26*; Δ*vib-1* and Δ*cre-1*; Δ*col-26*; Δ*vib-1* strains. (B) RT-PCR measurements of *clr-2* and *cbh-1* expression in the WT versus the Δ*col-26*, Δ*cre-1*, and the Δ*cre-1*; Δ*col-26*; Δ*vib-1* cultures after 5-days of growth on Avicel. Expression levels were normalized to WT. (C) The CMCase activity of Avicel cultures of WT versus Δ*col-26*, Δ*cre-1*, and the Δ*cre-1*; Δ*col-26*; Δ*vib-1* mutants during a time course of growth on Avicel.

Our data supported a role for CRE1 in CCR and a role for COL26 in the regulation of glucose utilization. We therefore tested sensitivity of the Δ*cre-1*; Δ*vib-1* and the Δ*col-26*; Δ*vib-1* mutants to AA. The Δ*cre-1*; Δ*vib-1* and the Δ*cre-1* mutants were both sensitive to AA (Figure 6B), indicating the Δ*cre-1* mutation is epistatic for CCR to Δ*vib-1*, while the Δ*col-26*; Δ*vib-1* mutant was insensitive to AA, consistent with the active CCR phenotype of the *col-26* and the Δ*vib-1* mutants. However, although CCR was impaired in the Δ*cre-1*; Δ*vib-1* mutant, the double mutant was still unable to produce cellulolytic enzymes and grow on Avicel (Figure 8A). Similar to the Δ*col-26* mutant, the Δ*col-26*; Δ*vib-1* mutant also showed defects in glucose consumption (Figure 7B). Although the Δ*col-26*; Δ*vib-1* mutant was unable to utilize Avicel, it showed slightly higher enzyme levels than that of the Δ*vib-1* mutant (Figure 8A). We therefore hypothesized that simultaneously preventing CRE1-mediated CCR and reducing glucose sensing/metabolism via inactivation of *col-26* would restore cellulase gene expression and enzyme activity in a Δ*vib-1* mutant. As predicted, a Δ*cre-1*; Δ*col-26*; Δ*vib-1* triple mutant utilized Avicel, produced significant cellulase activity and displayed a secretome similar to WT after 5 days of growth on Avicel (Figure 8A and S5). RT-PCR experiments from the Δ*cre-1*; Δ*col-26*; Δ*vib-1* Avicel cultures showed that expression levels of *clr-2* and *cbh-1* were restored in the triple mutant (Figure 8B).

**Figure 9:**
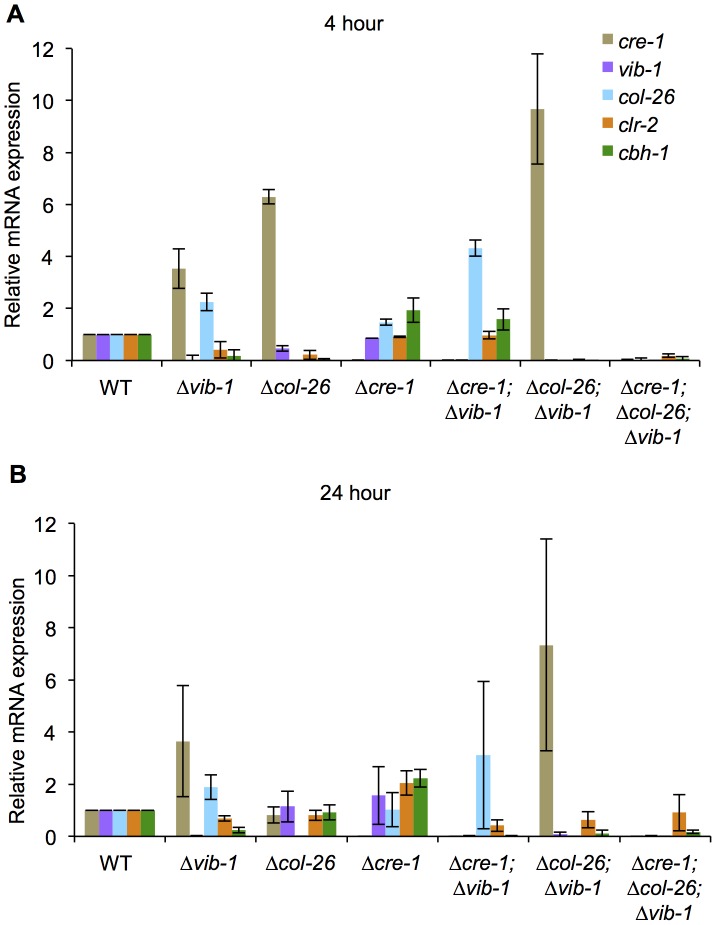
Suppression of *cre-1* and *col-26* expression by VIB1 plays a role in early inductive and utilization phase during growth on cellulose. The transcriptional expression of *cre-1*, *vib-1*, *col-26*, *clr-2*, and *cbh-1* were measured by RT-PCR at 4 hrs (A) and 24 hrs (B) after 16 hr sucrose growth cultures were transferred to Avicel conditions. Expression levels were normalized to WT.

Although simultaneous deletion of *cre-1* and *col-26* restored utilization of cellulose in the Δ*vib-1* mutant, a significant lag in growth and enzyme activity in the triple mutant was observed as compared to the WT, Δ*cre-1*, or Δ*col-26* mutants (Figure 8C). To assess whether the Δ*cre-1*; Δ*col-26*; Δ*vib-1* mutant was also delayed in transcriptional response upon exposure to cellulose, we measured expression levels of *clr-2*, *cbh-1*, *cre-1*, *vib-1* and *col-26* in the Δ*vib-1*, Δ*col-26*, Δ*cre-1*, Δ*cre-1*; Δ*vib-1*, Δ*col-26*; Δ*vib-1*, and Δ*cre-1*; Δ*col-26*; Δ*vib-1* mutants as compared to the WT strain at 4 hrs and 24 hrs after cultures were shifted to Avicel conditions. Consistent with the enzyme activity assay and growth phenotype (Figure 8C), induction of *clr-2* and *cbh-1* was delayed in the Δ*cre-1*; Δ*col-26*; Δ*vib-1* mutant (Figure 9). However, in the Δ*col-26* mutant at the 4 hr time point, expression levels of *cre-1* were significantly higher than in the Δ*vib-1* mutant, with the Δ*col-26*; Δ*vib-1* mutant showing an additive phenotype of significantly increased *cre-1* expression levels. At the 24 hr time point, expression levels of *cre-1* were only maintained in the Δ*vib-1* and Δ*col-26*; Δ*vib-1* mutants, but not in the Δ*col-26* mutant. These data suggest that COL26 may function to repress *cre-1* transcription to promote relief of CCR during the initial response to cellulolytic induction. Surprisingly, although the Δ*cre-1*; Δ*vib-1* mutant was unable to utilize cellulose, induction of both *clr-2* and *cbh-1* were near WT levels at the 4 hr time point, unlike the Δ*vib-1* mutant (Figure 9A). However, at the 24 hr time point, expression levels of *clr-2* were low and *cbh-1* was undetectable in Δ*cre-1*; Δ*vib-1* mutant (Figure 9B). These data suggest that although the Δ*cre-1*; Δ*vib-1* can respond to cellulolytic induction by increasing *clr-2* and thus *cbh-1* expression levels, induction signaling cannot be maintained, perhaps due to repression by COL26 or by other factors present/absent in a Δ*vib-1* mutant background. The fact that the *Δcre-1*; *Δcol-26*; Δ*vib-1* mutant does not show WT restoration of initial cellulolytic induction (Figure 8C; Figure 9A) supports the hypothesis that additional unknown factors remain to be identified that play a role in nutrient sensing/signaling and the regulation of cellulose utilization in *N. crassa*.

## Discussion

In this study, we showed that a Δ*vib-1* mutant displayed severe growth defects on cellulose, which was correlated with a lack of cellulolytic enzyme activity. By using RNA-seq data, we showed that expression of the Avicel regulon was significantly decreased in the Δ*vib-1* mutant, a phenotype that was rescued by constitutive expression of *clr-2*. Induction of *clr-2* is dependent upon a signal cascade from cellobiose or derivative and functional CLR1 (Figure 1) [8]. Here we showed that VIB1 is not involved in inducer signal processing or perception because the Δ3βG; *Δvib-1* mutant produced cellulolytic enzymes in response to cellobiose. These data indicated that VIB1 functions upstream of regulators that mediate inducer-dependent signal transduction and cellulase gene expression and activity.

Our transcriptional profiling revealed that, under Avicel conditions, a deletion of *vib-1* led to an increase in transcription of genes in metabolism and energy as well as genes reported to mediate CCR. These results suggested that cellulolytic induction was mis-regulated in the Δ*vib-1* mutant. In the presence of glucose, *N. crassa* adjusts its metabolism for a high rate of glycolysis and directs carbon flux to respiration and fermentation for biosynthesis and energy production [58], while genes involved in utilization of alternative carbon sources are repressed in a CRE1-dependent manner [26], [27]. When lignocellulose is the only carbon source, CCR is relieved to allow the synthesis of “scouting” enzymes that liberate inducer molecules, such as cellobiose [9], [44], [59]. In *S. cerevisiae*, glucose is sensed through a multifaceted mechanism including direct detection of glucose by glucose receptors/transporters on the plasma membrane and by the sensing of glucose-6-P and other metabolites by metabolic enzymes. The glucose signals are transmitted to CCR mainly through the Snf1 complex and the Mig1 (CreA/Cre1 homolog) transcriptional repressor complex [60], [61]. In *A. nidulans*, mutations in two hexose kinase genes (*hxkA/glkA4*) results in inappropriate de-repression of genes under glucose growth conditions, although to a lesser extent than a *creA* mutant strain [29]. Here we show that simply eliminating CRE1-mediated CCR did not rescue the growth defect of Δ*vib-1* mutant on Avicel, but that a deletion of *col-26* was also required.

**Figure 10:**
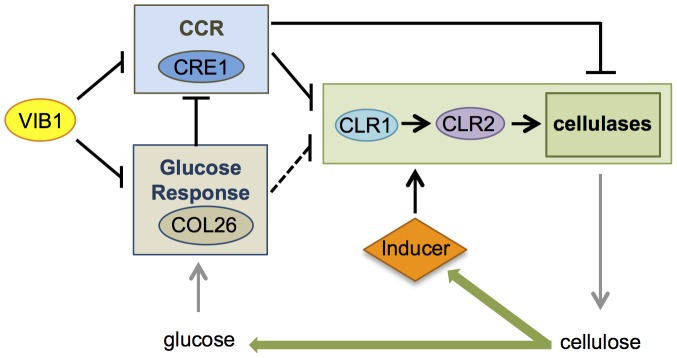
Model for role of VIB1 in regulating glucose sensing/metabolism and CCR under cellulolytic conditions. In an early encounter of *N.* crassa to cellulose, “scouting” enzymes induced by carbon starvation act on cellulose to liberate cellulolytic inducers that activate signaling cascades that include activation of CLR1, and subsequent expression of CLR2 and induction of genes encoding cellulases. Efficient cellulolytic induction also requires de-repression of carbon catabolite repression (CCR), as many cellulase genes are not expressed even in the presence of an inducer if a preferred carbon source is available. In the early cellulolytic induction, VIB1 functions to repress CRE1-mediated CCR and glucose sensing/metabolism by COL26, which results in CCR de-repression and productive cellulolytic responses. COL26 also plays a role in repressing *cre-1* expression and thus alleviating CCR. As cellulases are produced, glucose is liberated from cellulose and *N. crassa* transits into the utilization phase, which is associated with reduced transcription of cellulases [44]. Repressive function of VIB1 on *col-26* expression may be important for tuning cellular responses for the need to produce sufficient enzymes to liberate simple sugars from cellulose, but without over-activating CCR. VIB1 also likely regulates other factors important for cellulolytic induction.

The *Δcol-26* mutant exhibited a growth defect on glucose, fructose and sucrose, which was not associated with a deficiency in glucose transport (Figure 7B). In *T. reesei*, a strain carrying a mutation in *bglR* shows reduced expression of β-glucosidase genes, suggesting the BglR plays a positive role in CCR by increasing glucose release from cellobiose [15]. However, our analyses of cellulolytic activity of secreted enzymes in the *Δcol-26* mutant showed no difference in glucose versus cellobiose release (Figure 8A), a result that is in contrast to the strongly reduced glucose release from culture supernatants in the *Δ*3βG mutant (which lacks extracellular β-glucosidase activity) (Figure S2). Although we have not determined how glucose metabolism is changed in the Δ*col-26* mutant, the resistance of Δ*col-26* to 2-DG inhibition suggests a defect in glucose sensing/metabolism; CRE1-mediated CCR was still functional (as shown by insensitivity to AA). The fact that a deletion of *col-26* and *cre-1* restored growth of Δ*vib-1* on Avicel suggests a synergistic effect between glucose sensing/metabolism mediated by COL26 and CRE1-regulated CCR in repressing cellulolytic induction (Figure 10). However, other unknown factors in addition to CRE1 and COL26 play a role in the Δ*vib-1* mutant, because the Δ*cre-1*; Δ*col-26*; Δ*vib-1* mutant showed a significant lag in gene induction and enzyme secretion under cellulolytic conditions (Figure 8C). Future experiments to identify additional mutations that fully suppress the Δ*vib-1* cellulolytic phenotype and the identification of direct targets of VIB1 will be most informative for further dissection of glucose sensing and CCR in filamentous fungi.

Our data supports the model that the regulatory function of VIB1 on CRE1-mediated CCR and COL26-mediated glucose sensing/metabolism functions during different stages of the cellulolytic response (Figure 10). At induction stage, both VIB1 and COL26 negatively regulate CRE1-mediated CCR (Figure 9), thus allowing a relief of CCR and efficient induction of cellulolytic genes in response to cellulose. During the utilization phase, glucose is released from cellulose, and glucose sensing/signaling via COL26 may repress cellulolytic responses, with VIB1 functioning to dampen this inhibition. As many cellulolytic genes are subject to carbon catabolite repression and a requirement for CLR2 for induction, the cellular response to plant biomass may depend on the relative strength of these two antagonizing forces (Figure 10). Mechanistically, how VIB1 exerts its function on glucose sensing/metabolism via COL26 and CCR via CRE1 remain to be elucidated.

In the hyper-secreting *T. reesei* strain, RUT-C30, disruption of phosphoglucose isomerase gene (*pgi1*) blocks formation of fructose-6-P from glucose-6-P and increased cellulase production on glucose. This increase relies on a genetic interaction between the Δ*pgi1* mutation and the *cre1-1-1* mutation in the RUT-C30 background [62]. Interestingly, both the hyper-secreting *T. reesei* RUT-C30 and PC-3-7 strains have mutations in *cre1* and *bglR/col-26* [15], [63], [64], but whether a synergy exists between Δ*cre1* and Δ*bglR* in *T. reesei*, as in *N. crassa*, and its relationship to *T. reesei vib1* is unclear. Many cellulolytic enzyme hyper-producers such as *T. reesei* RUT-C30 and PC-3-7, and *P. decumbens* JU-A10-T show relief from CCR, but contain a large number of mutations in additional genes that contribute to the hyper-production phenotype [2], [15], [63]–[65]. Identifying and characterizing possible synergistic effects of the different mutations on hyper-production of lignocellulose enzymes, as shown in this study, will be a challenge.

The function of VIB1 in regulating glucose sensing/metabolism and CCR plays a role in the utilization of other complex substrates. VIB1 is required for extracellular protease production in response to carbon and nitrogen starvation, a function shared by its homolog in *A. nidulans*, *xprG* [66]–[70]. The Δ*vib-1* mutant also exhibits inappropriate temporal and spatial conidiation and has defects in protoperithecia formation [48], [70], two developmental events that are regulated by nitrogen and glucose limitation and signaling [71]. A shotgun proteomic analysis of culture supernatant of the P*vib-1* strains under carbon source depletion showed a higher amount of intracellular proteins relative to WT (Table S3). These data are in consistent with a role of VIB1 in promoting cell death [47], [48], [72], and in autolysis in *A. nidulans* [73], perhaps via perturbed nutritional signaling. Autolysis is frequently observed in submerged batch cultures in industrial bioprocessing, and promotes cryptic growth for survival and protein production under nutrient-depleted conditions [74]. Further manipulations of *vib-1* and its homologs in filamentous fungi may yield economic benefits via the regulation of autolysis under industrial settings.

In summary, our data show that VIB1 is an essential regulator for cellulase production under inductive conditions and identifies COL26 as an important player in glucose sensing/metabolism. As VIB1 mediates metabolic changes as well as programmed death, two properties shared by mammalian tumor suppressor p53 [75], [76], the molecular mechanism in linking the two could be conserved, and further investigation of *vib-1* function and its homologs in filamentous fungi may also shed light on cancer research.

## Materials and Methods

### Strains

FGSC 2489 was used as the WT reference strain and background for mutant strains [46]. FGSC 11308 (*Δvib-1*; *mat a*), FGSC 11309 (*Δvib-1*; *mat A*), FGSC 11030 (*Δcol-26*; *mat a*), FGSC 11031 (*Δcol-26*; *mat A*) were obtained from the Fungal Genetics Stock Center (http://www.fgsc.net/) [50]. The *vib-1* mis-expression strain P*vib-1* (P*vib-1*; Δ*vib-1*) was constructed by transforming FGSC 11308 with a DNA fragment containing the promoter of the clock controlled gene 1 (*ccg-*1) and the open reading frame and 3′ untranslated region (UTR) of *vib-1* and homologous and flanking regions from the coding sequence of the *his-3* gene. Transformants were selected for histidine prototrophy [77] and backcrossed to FGSC 2489 to obtain a *his-3::pccg-1-vib-1*; Δ*vib-1* homokaryotic strain. The *Tr vib1* mis-expression strain P*Tr-vib1* (P*Tr vib1*; Δ*vib-1*) was created in the same way except that the open reading frame and 3′UTR of *Tr vib1* was used. The Pc *clr-2*, the *Δcre-1*, the Δ3βG and the Δ3βG *Δcre-1* strains were from previous studies [9], [10], [26]. The Pc *clr-2*; *Δvib-1* strain, the Δ3βG; *Δvib-1* strain, the *Δcol-26*; *Δvib-1* strain, the *Δcre-1*; *Δcol-26* strain, and the Δ*cre-1*; Δ*col-26*; Δ*vib-1* strain were created through crosses.

### Culture conditions

*N. crassa* cultures were grown on Vogel's minimal medium (VMM) [78]. Unless noted, 2% (w/v) sucrose was used as a carbon source. Strains were pre-grown on 3 mL VMM slants at 30°C in dark for 24 hrs, then at 25°C in constant light for 4–10 days to stimulate conidia production. For flask cultures, conidia were inoculated into 100 mL of liquid media at 10^6^ conidia/mL and grown at 25°C in constant light and shaking (200 rpm). To test 2-DG and allyl alcohol sensitivity, 3 mL of liquid media containing either 0.2% (w/v) 2-DG (Sigma Aldrich, MO) or 100 mM allyl alcohol were inoculated with 10^6^ conidia/mL and grown in 24-well plates at 25°C in constant light and shaking (200 rpm).

For crosses, one parental strain was grown on synthetic crossing medium [79] as the female for 2 weeks at room temperature for protoperithecial development. The other parental strain was used as the male to fertilize the protoperithecia. Crosses were kept for 3 weeks at room temperature. Ascospores were collected and activated as described [80], plated on 1% VMM, and incubated at room temperature for 18 hrs. Germinated ascospores were selected and transferred to selective slants for further screen and confirmation.

### Media shift experiments

Cultures were grown on sucrose for 16 hrs, centrifuged at 2000 g for 10 min and washed in VMM or MM without a carbon source, followed by 4 hrs growth in 100 mL VMM or MM with 2% carbon source (sucrose, cellobiose, Avicel PH-101 (Sigma Aldrich, MO)) or with no carbon source added.

### RNA preparation and qRT-PCR analysis

Mycelia were harvested by filtration and flash frozen in liquid nitrogen. RNA was extracted using the Trizol method (Invitrogen) and further purified using RNeasy kits (QIAGEN). Four ng of RNA was used as template in each quantitative RT-PCR (qRT-PCR) reaction. qRT-PCR was carried out using EXPRESS One-Step SYBR GreenER kit (Invitrogen) and Applied Biosystems Step One Plus Real Time PCR system. qRT-PCR were done in biological duplicates or triplicates with actin as the endogenous control. Relative expression levels were normalized to actin, and fold changes in RNA level were the ratios of the relative expression level on inducing conditions to no carbon conditions.

### RNA sequencing and transcription expression analysis

Libraries were prepared according to standard protocols from Illumina Inc (San Diego, CA) and sequenced on the HiSeq 2000 platforms at QB3 Vincent J. Coates Genomics Sequencing Laboratory (CA). Sequenced reads were mapped against predicted transcripts from the *N. crassa* OR74A genome [81](*Neurospora crassa* Sequencing Project, Broad Institute of Harvard and MIT http://www.broadinstitute.org/) with Tophat v2.0.4 [82]. Transcript abundance (FPKM) was estimated with Cufflinks v2.0.2 mapping against reference isoforms and differential gene expression were analyzed with Cuffdiff v2.0.2 [83]. Biological replicates used for RNA-seq showed high reproducibility. The Pearson correlation of FPKM on log basis (p-value<2.2e-16): r_p_≥0.96 between WT (Nc) replicates, r_p_≥0.91 between WT (Av) replicates, r_p_≥0.99 between Δ*vib-1* (Nc) replicates, and r_p_≥0.96 between Δ*vib-1* (Av) replicates.

For hierarchical clustering analysis, FPKM were log transformed, normalized and centered on a per gene basis with Cluster 3.0 [84] so that values from each gene ranged from −1 (minimum) to 1 (maximum). Average linkage clustering was performed with Euclidean distance as the similarity metric. Functional category analysis was done as described in [8]. Lists of genes were matched against the MIPS Functional Category Database [85], and significance of enrichment was calculated.

### Enzyme activity assays

For CMCase and xylanase activity assays, Azo-CM-Cellulose and Azo-xylan (Beechwood) from Megazyme (Wicklow, Ireland) were used as substrates. Protein concentration was measured with the Bradford assay (BioRad). Cellulase assays were conducted by mixing 500 µL of culture supernatant with 500 µL 0.5% (w/v) Avicel in 100 mM sodium acetate, pH 5.0, and incubated with shaking at 37°C for 5 hrs. Reactions were stopped by centrifugation at 2000 g for 5 min and by addition of 9 volumes of 0.1 M NaOH to the reaction supernatants. Released glucose and cellobiose were separated on a PA-200 HPAEC column and analyzed on Dionex ICS-3000 as described in [45].

### Glucose uptake assays

Strains were grown in 3 mL VMM with 2% cellobiose as the carbon source in the well of 24-well plates at 25°C in constant light with shaking (200 rpm) for 40 hrs to reach the same mycelial biomass, then glucose was added into each culture such that the culture was grown in MM with 1% (w/v) glucose for 1 hr. The cultures were thoroughly washed with MES buffer (10 mM 2-(N-morpholino)ethanesulfonic acid, 100 mM NaCl), and each washed culture was transferred into 4 ml of MES buffer supplemented with 10 mM glucose and grown at room temperature for 1 hr with shaking at 550 rpm. Culture supernatants were sampled at 5, 20, and 60 min, and diluted in 50 volumes of 0.1 M NaOH. Glucose levels were measured using Dionex ICS-3000 HPAEC-PA 200 and MES buffer instead of VMM was used to avoid precipitation that interferes with downstream analysis.

### Protein gel electrophoresis

Culture supernatants were mixed with 4× SDS loading buffer and boiled for 10 min before loading onto Criterion 4–15% Tris-HCl Precast Gel (Bio-Rad). GelCode Blue Stain Reagent (Thermo Scientific) was used for gel staining.

### Microscopy and imaging

Strains were inoculated in 2% sucrose VMM and grown at 25°C for 12 hrs in eight-chamber Lab-Tek chambered cover glass (Nalge Nunc International, Naperville, IL). Localization of VIB1-GFP was observed using a 100×1.4 NA oil immersion objective on a Leica SD6000 spinning disk confocal with 488 nm laser and controlled by Metamorph software. Z-series stacks were collected and maximum intensity projections were created using ImageJ. For medium shift experiment, the cultures in the chamber were washed with VMM without carbon sources and VMM with 0.5% Avicel was added, followed by immediate time-lapse recordings with an interval of 15 min.

### Proteomic analysis

Equal volume of culture supernatants of WT and P*vib-1* strains was subjected to SDS-PAGE and secretome proteins identified as described in [86]. In-gel trypsin-digestion was performed according to manufacture protocol (Promega, Trypsin Gold). Digested peptides were separated using ProtID-Chip-43 (II) and analyzed using the Agilent 6510 Q-TOF LC/MS as in [9].

## Supporting Information

Figure S1 VIB1 functions in xylanase production and localizes to nuclei in both sucrose and cellulose media. (A) The Δ*vib-1* mutant grew slowly on xylan and showed reduced xylanase activity. (B) Mycelial biomass accumulation after 24 hrs of growth in the Δ*vib-1* mutant as compared to WT in VMM containing 2% (w/v) of glucose, cellobiose, or xylose as the sole carbon source. (C) Fluorescence microscopy showing localization of VIB1-GFP to nuclei under both sucrose and Avicel conditions.
(TIFF)

Figure S2 Mutations in Δ3βG genes rescue the cellulase-deficient phenotype of the Δ*vib-1* mutant on Avicel. Cellulase activity in the Δ3βG; Δ*vib-1* mutant compared to the WT, Δ*vib-1* and the Δ3βG strains after 4 days of growth on 2% Avicel. Cellulase activity was measured using Avicel as substrate (see Materials and Methods).
(TIFF)

Figure S3 Secreted protein levels and cellulase activity in the Δ*cre-1*, Δ*creB*, Δ*creD*, and Δ*col-26* mutants relative to WT and the Δ*vib-1* mutant. (A) The Δ*cre-1*, Δ*creB*, Δ*creD* and Δ*col-26* mutants in comparison to WT and the Δ*vib-1* mutant were screened for secreted protein levels after 7 days of growth on 2% Avicel. (B) CMCase activity of the Δ*cre-1*, Δ*creB*, Δ*creD* and Δ*col-26* mutants in comparison to WT and the Δ*vib-1* mutant after 7 days of growth on 2% Avicel.
(TIF)

Figure S4 *clr-2* expression levels in the Pc *clr-2* strains as compared to WT and the Δ*vib-1* mutant. *clr-2* expression levels at 4 hrs after a shift of 16 hr old sucrose-grown cultures to Avicel were measured in WT, Δ*vib-1*, Pc *clr-2*, and Pc *clr-2*; Δ*vib-1* by RT-PCR and normalized to the WT level.
(TIFF)

Figure S5 The Δ*cre-1*; Δ*col-26*; Δ*vib-1* mutant displays a cellulose secretome similar to WT. WT, Δ*vib-1*, Δ*cre-1*, Δ*col-26*, Δ*cre-1*; Δ*vib-1*, Δ*col-26*; Δ*vib-1*, and Δ*cre-1*; Δ*col-26*; Δ*vib-1* strains were inoculated with 10^6^ conidia/ml and grown on Avicel for 5 days. The culture supernatants were subsequently separated by SDS-PAGE.
(TIFF)

Table S1 Gene list of the 91 Avicel-regulon genes differentially expressed in Δ*vib-1* as compared to WT on Avicel.
(XLSX)

Table S2 Gene list of the 770 genes differentially expressed between no carbon and Avicel conditions in Δ*vib-1*.
(XLSX)

Table S3 Intracellular *N. crassa* proteins enriched in the supernatant of P*vib-1* cultures relative to WT after carbon depletion.
(XLSX)
