# Supplementary figures and images for: VIB1, a Link between Glucose Signaling and Carbon Catabolite Repression, Is Essential for Plant Cell Wall Degradation by Neurospora crassa
Source: PLoS Genet. 2014 Aug 21;10(8):e1004500. doi: 10.1371/journal.pgen.1004500 (PMC4140635; doi:10.1371/journal.pgen.1004500)

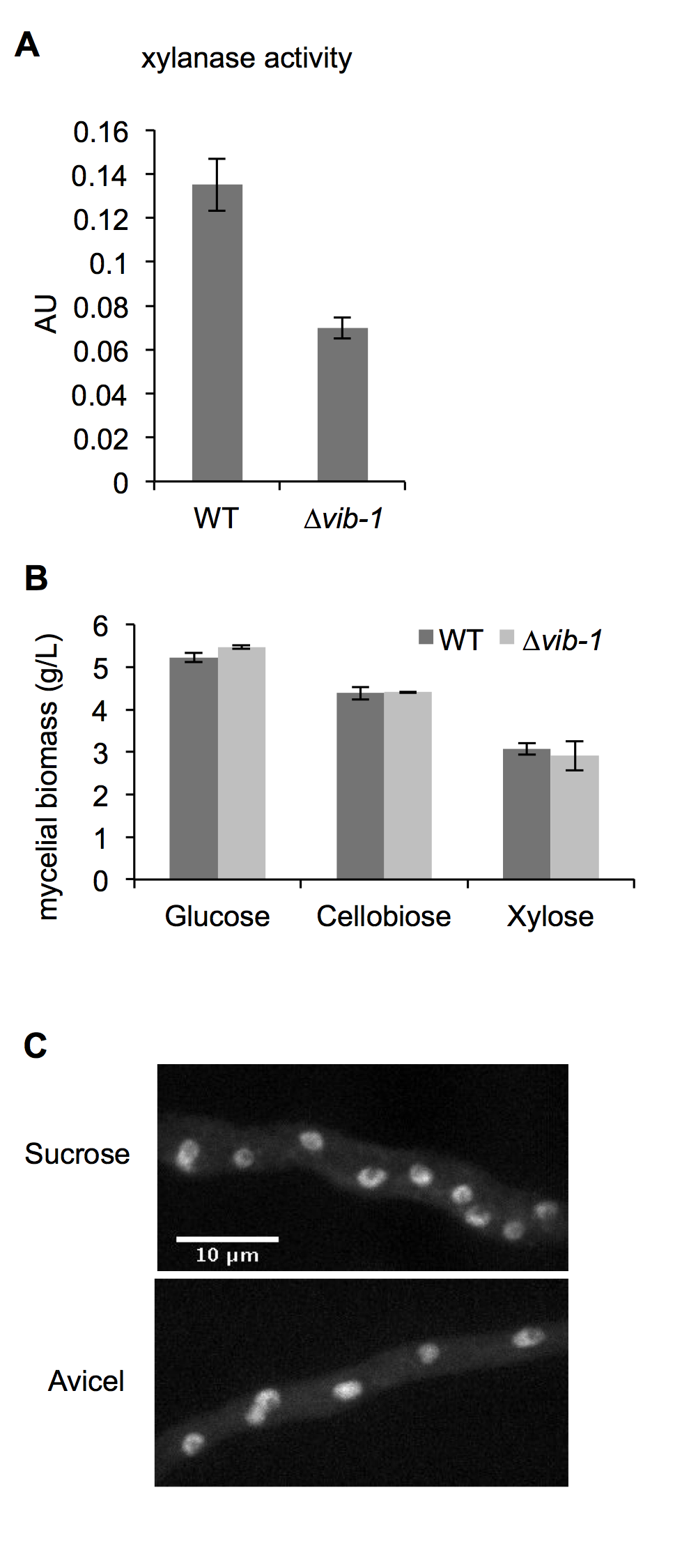

Supplement: Figure S1 — VIB1 functions in xylanase production and localizes to nuclei in both sucrose and cellulose media. (A) The Δvib-1 mutant grew slowly on xylan and showed reduced xylanase activity. (B) Mycelial biomass accumulation after 24 hrs of growth in the Δvib-1 mutant as compared to WT in VMM containing 2% (w/v) of glucose, cellobiose, or xylose as the sole carbon source. (C) Fluorescence microscopy showing localization of VIB1-GFP to nuclei under both sucrose and Avicel conditions. (TIFF) [file pgen.1004500.s001.tiff]

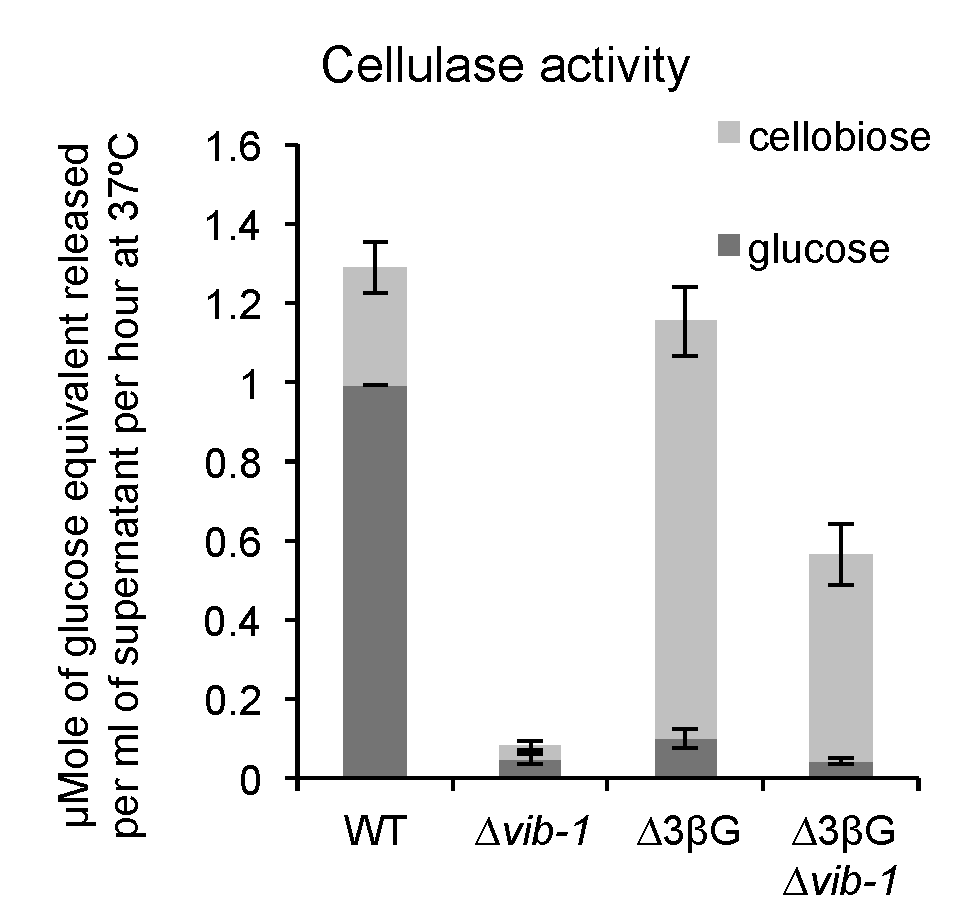

Supplement: Figure S2 — Mutations in Δ3βG genes rescue the cellulase-deficient phenotype of the Δvib-1 mutant on Avicel. Cellulase activity in the Δ3βG; Δvib-1 mutant compared to the WT, Δvib-1 and the Δ3βG strains after 4 days of growth on 2% Avicel. Cellulase activity was measured using Avicel as substrate (see Materials and Methods). (TIFF) [file pgen.1004500.s002.tiff]

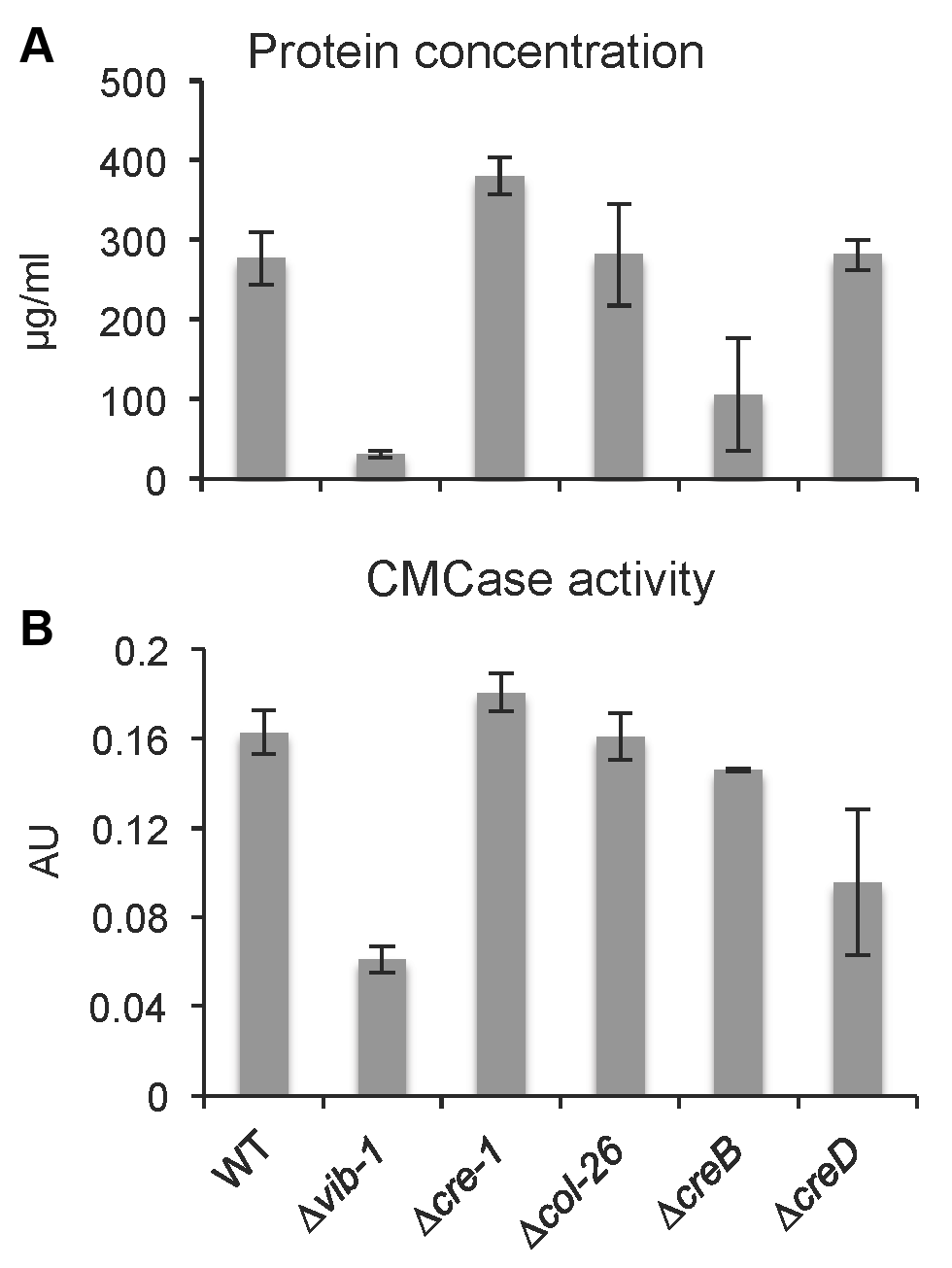

Supplement: Figure S3 — Secreted protein levels and cellulase activity in the Δcre-1, ΔcreB, ΔcreD, and Δcol-26 mutants relative to WT and the Δvib-1 mutant. (A) The Δcre-1, ΔcreB, ΔcreD and Δcol-26 mutants in comparison to WT and the Δvib-1 mutant were screened for secreted protein levels after 7 days of growth on 2% Avicel. (B) CMCase activity of the Δcre-1, ΔcreB, ΔcreD and Δcol-26 mutants in comparison to WT and the Δvib-1 mutant after 7 days of growth on 2% Avicel. (TIF) [file pgen.1004500.s003.tif]

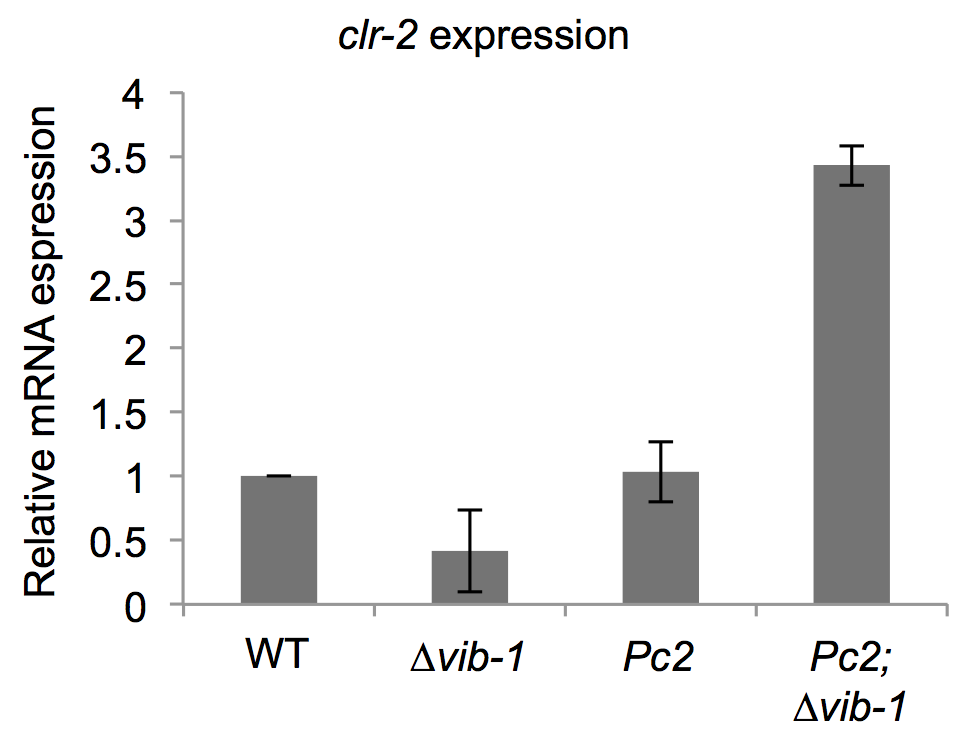

Supplement: Figure S4 — clr-2 expression levels in the Pc clr-2 strains as compared to WT and the Δvib-1 mutant. clr-2 expression levels at 4 hrs after a shift of 16 hr old sucrose-grown cultures to Avicel were measured in WT, Δvib-1, Pc clr-2, and Pc clr-2; Δvib-1 by RT-PCR and normalized to the WT level. (TIFF) [file pgen.1004500.s004.tiff]

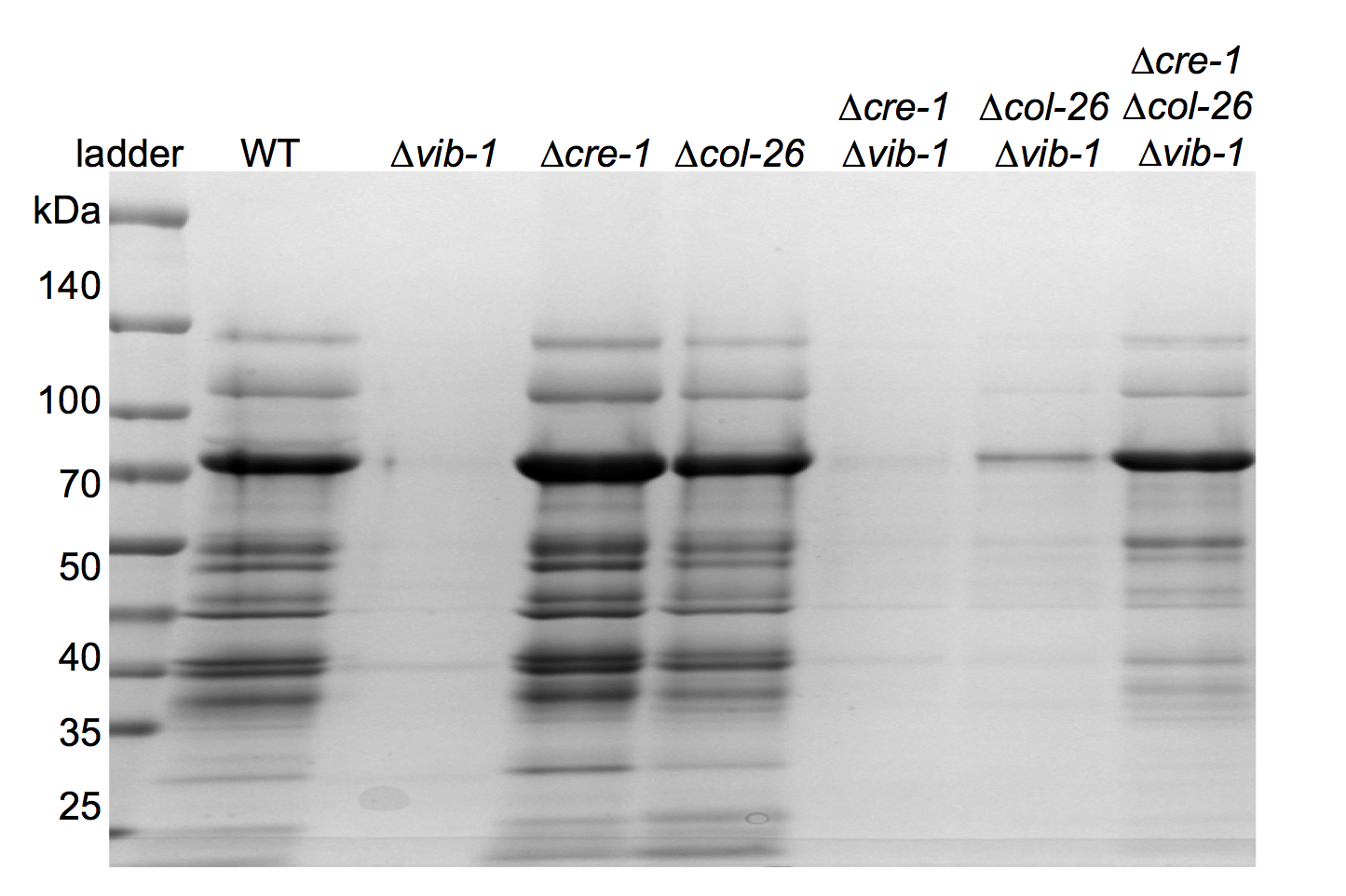

Supplement: Figure S5 — The Δcre-1; Δcol-26; Δvib-1 mutant displays a cellulose secretome similar to WT. WT, Δvib-1, Δcre-1, Δcol-26, Δcre-1; Δvib-1, Δcol-26; Δvib-1, and Δcre-1; Δcol-26; Δvib-1 strains were inoculated with 106 conidia/ml and grown on Avicel for 5 days. The culture supernatants were subsequently separated by SDS-PAGE. (TIFF) [file pgen.1004500.s005.tiff]
